# Recent advances in acid sensing by G protein coupled receptors

**DOI:** 10.1007/s00424-024-02919-y

**Published:** 2024-02-10

**Authors:** Maike D. Glitsch

**Affiliations:** Medical School Hamburg, Am Sandtorkai 1, 20457 Hamburg, Germany

**Keywords:** Proton-sensing receptors, OGR1, GPR68, TDAG8, GPR4, GPR65

## Abstract

Changes in extracellular proton concentrations occur in a variety of tissues over a range of timescales under physiological conditions and also accompany virtually all pathologies, notably cancers, stroke, inflammation and trauma. Proton-activated, G protein coupled receptors are already partially active at physiological extracellular proton concentrations and their activity increases with rising proton concentrations. Their ability to monitor and report changes in extracellular proton concentrations and hence extracellular pH appears to be involved in a variety of processes, and it is likely to mirror and in some cases promote disease progression. Unsurprisingly, therefore, these pH-sensing receptors (pHR) receive increasing attention from researchers working in an expanding range of research areas, from cellular neurophysiology to systemic inflammatory processes. This review is looking at progress made in the field of pHRs over the past few years and also highlights outstanding issues.

## Introduction

Acids are defined as chemicals that give off protons, and acid sensing therefore involves proton sensing. Proton concentrations are converted into pH values and generally, blood pH in a healthy individual is taken to be pH 7.4 (equivalent to approximately 40 nM protons). Deviations as small as ± 0.05 pH units are considered pathological states of acidosis or alkalosis, respectively. For a long time, the prevailing dogma was that interstitial tissue pH was equivalent to blood pH, and that extracellular pH did not fluctuate in any physiologically meaningful manner. Rather, deviations from physiological pH values were taken to be long-term consequences of pathological events. We now know that interstitial tissue pH deviates from and tends to be lower than blood pH (e.g. brain, skin, see below) and that interstitial pH can fluctuate acutely as well as in the long term.

A number of physiological processes involve acidification of the interstitial fluid of the tissue in question. Bone metabolism, specifically bone resorption, is contingent on extracellular acidification [71], and the respiratory burst of immune cells is accompanied by proton extrusion [83], leading to acidification of the affected tissue. Other examples include luminal acidification of the epididymis during sperm maturation and storage [79], cyclic changes in extracellular pH for proper enamel formation during tooth development [39] and acidification of the synaptic cleft during synaptic transmission [80]. This list is not exhaustive and just serves to illustrate the variety of distinct physiological processes that are contingent on or affected by extracellular acidification. Importantly, extracellular acidification occurs to different extents and over a range of distinct time scales, depending on the process (ms to days). Finally, it is well established that virtually all pathological processes (including cancer, inflammatory conditions, trauma, stroke) are accompanied by sometimes dramatic changes in tissue pH [23]. Changes in extracellular pH are sensed by proteins that change their activity in an extracellular pH-dependent manner. Such receptors are called proton-sensing receptors since their conformational change and thus altered activity state is contingent on proton-binding. Whilst physiological fluctuations in extracellular pH bring about changes in proton-sensing receptor activity (patterns) that are required to allow physiological process to go ahead (or do not result in activation of proton-sensing receptors, thus effectively going about unnoticed by the surrounding cells), atypical proton-sensing receptor activation can be argued to be part of pathologies. In fact, it tends to exacerbate disease progression and symptoms, and in some cases even be a (leading) cause of the pathology.

Research into proton-sensing receptors has increased dramatically over the past 25 years, though it is still modest compared to other fields with an annual maximum (to date) of 58 publications in 2021 (PubMed search using the key word “proton sensing receptor”). Whilst virtually all proteins exhibit allosteric pH sensitivity, proton-sensing receptors are activated by extracellular protons and broadly fall into two categories: G protein coupled receptors (GPCRs) and ion channels. The emphasis of this review will be on GPCRs that are activated by extracellular protons because, in 2020 alone, these receptors accounted for 62.5% of all publications on proton-sensing receptors. This review adopts the view taken by Imenez Silva and Wagner and considers GPCRs that are activated (as opposed to allosterically modulated) by protons as proton-activated rather than proton-sensitive receptors [25]. These proton-activated GPCRs, whose activation state is, by definition, dependent on extracellular pH, will be abbreviated with the acronym pHR.

pHRs are class A GPCRs that consist of three members, G protein coupled receptor 4 (GPR4), T cell death-associated gene 8 (TDAG8, aka GPR65) and ovarian cancer G-protein coupled receptor 1 (OGR1, aka GPR68) [25]. They are all at least partially active at pH 7.4 [25], suggesting a basal level of activity even in tissues and cells that do not require pH fluctuations for their normal function. Importantly, OGR1 has been shown to be co-activated by protons and actin polymerisation state of the cells in which it is expressed. Hence, an increase in proton concentration is not necessarily sufficient to activate this receptor, but both a certain extracellular proton concentration as well as a certain level of actin polymerisation need to be achieved for OGR1 to be active [90]. This may have important implications for our understanding of its physiological (and pathological) roles, as discussed below.

Careful monitoring of extracellular pH is likely important for any cell, given the powerful modulatory impact that protons can have on protein structure and hence function. Furthermore, protons are metabolites that can be extruded from cells, for example in a bid to maintain intracellular pH at a desired level. pHRs may therefore also play a role in sensing overall health and viability of cells and tissues whose function does not depend on or is accompanied by physiological fluctuations in extracellular pH.

There have been numerous excellent reviews on pHRs in recent years [23, 25, 81], which is why this review will focus on papers published from 2020 onwards. It will consider notable developments in our understanding of physiological and pathological roles for pHRs in a tissue- or process-dependent manner. The acronyms OGR1, GPR4 and TDAG8 will be used throughout this review, even when the original publication uses GPR68 (for OGR1) and/or TDAG8 (for GPR65).

## Nervous system

Measurements of extracellular pH in the human brain are difficult to obtain and most techniques yield values that have been averaged over space and time, and which therefore do not account for acute fluctuations in small and limited areas such as the synaptic cleft. The picture is further complicated because the extracellular fluid of the brain is composed of cerebrospinal fluid (largely derived from the choroid plexus) and interstitial fluid of the brain parenchyma. It is therefore likely that different areas of the brain experience different extracellular pH values. Nonetheless, it is established that extracellular brain pH is lower than blood pH (e.g. [35]), which means that pHRs are likely to be active at all times and that their activity pattern has the potential to mirror and reflect brain activity. Unsurprisingly, therefore, research into roles for pHRs in the brain under physiological and pathological conditions has increased over the past few years.

OGR1 is found on all neuronal compartments including most spines [95], and it is involved in learning and memory formation including emotional learning [95]. Furthermore, OGR1 has been implicated in hypothalamic regulation of food intake where inhibition of OGR1 function has orexigenic effects, suggesting that OGR1 promotes food intake [59]. Both findings would suggest a life-promoting role for OGR1 in the CNS under physiological conditions.

In cortical slice preparations from *OGR1*, *GPR4* or *TDAG8* knock-out (ko) mice, acidotic conditions only caused significantly increased neuronal damage in *OGR1* ko-derived slices compared to slice preparations from wildtype (wt) mice, suggesting a unique role for OGR1 in ischaemia [89]. Hence, OGR1 has been investigated in the context of stroke by surgically inducing transient middle cerebral artery occlusion (tMCAO) in male wt and *OGR1* ko mice [87, 89, 103]. In *OGR1*-deficient mice, the infarction volume was significantly increased and motor performance was more compromised than in wt mice [89]. A follow-on study then revealed a correlation between *OGR1* deletion and infarction size under conditions of mild but not severe haemorrhagic transformation (a break-down of the blood brain barrier that exacerbates the effect of ischaemia) [87]. Finally, RNA sequencing found that transient stroke induction increased expression levels of GPR4 and TDAG8 but not OGR1, and *OGR1* deletion did not affect expression levels of GPR4 or TDAG8 under control or ischaemic conditions, suggesting that OGR1 does not affect GPR4 or TDAG8 expression [103]. Results from this study also indicate that OGR1 may affect chaperone function and play a role in haemoglobin-mediated antioxidant mechanisms [103].

In a murine ischaemia reperfusion model using functional *TDAG8* ko by employing a transposon approach to disrupt *TDAG8* function rather than deleting the *TDAG8* gene, resulted in increased motor deficits and larger infarction size compared to wt mice [72]. Importantly, this study compared TDAG8 expression levels in the ipsi- and contralateral brain hemispheres and found a significant increase in TDAG8 expression in wt mice following tMCAO, likely due to increased microglia activity in the injured hemisphere, whilst expression levels of OGR1 and GPR4 remained unchanged [72]. Moreover, neither *OGR1* nor *GPR4* ko influenced infarction size compared to wt [72], which is in contradiction to results obtained in [89] and [87]. Future studies will have to address this apparent conflict, especially given that genetic background and method of inducing ischaemia were basically identical. Notably, the TDAG8 study focussed on male mice only and hence sex-dependent differences may be important here.

TDAG8 has also been implicated in neuroprotective effects of delayed chronic acid postconditioning (DCAP) following a stroke event [19]. In a photothrombotic stroke model, *TDAG8* ko resulted in higher levels of pro-inflammatory microglia and reduced expression of markers for axon growth and regeneration despite DCAP, pointing towards an involvement of TDAG8 in at least some of the beneficial treatment outcomes following DCAP [19].

Finally, TDAG8 has been implicated in freezing responses in mice [91]. In contrast to wt, *TDAG8* ko mice showed decreased freezing and increased explorative behaviour in response to low pH stimulation of the subfornical organ area, suggesting that TDAG8 may play a role in anxiety [91]. The subfornical organ is part of the circumventricular organs that lack a blood brain barrier. Hence, the subfornical organ is emerging as another central structure that elicits behaviours in response to blood pH changes, in addition to GPR4-expressing retrotrapezoid nuclei that promote breathing under conditions of acidosis [38]. This freezing response reflects sympathetic activation and may provide a time window in which an individual can assess a perceived threat before deciding on the most appropriate response [49].

GPR4 has been linked to two neurodegenerative conditions, Alzheimer’s disease and Parkinson’s disease. The link to Alzheimer’s disease was established in a transcriptome-wide association study that identified *GPR4* as a potential predictive biomarker, most likely due to pro-inflammatory effects on the immune system [102]. In a chemically induced mouse model of Parkinson’s disease, lack of GPR4 expression was shown to be neuroprotective in dopaminergic neurons, and pharmacological inhibition of GPR4 not only reduced neuronal apoptosis but also improved motor and memory functions [21]. Two additional studies link GPR4 to neuronal apoptosis [20, 94]; however, it remains unclear whether and how results obtained in these studies that were conducted on isolated cells translate into tissue context. Hence, GPR4 may be involved in neurodegenerative processes and its activity appears to be detrimental to neuronal health.

## Vasculature

One of the many distinct roles of vasculature is to ensure that carbon dioxide (CO_2_), a metabolic byproduct, can be transported from tissues to the lung where it can be eliminated from the human body. CO_2_ also combines with water to produce carbonic acid, and excess acid can be removed from the blood stream in the kidneys through excretion of protons into the urine. Lack of removal of CO_2_ due to compromised, insufficient or inadequate vascularisation therefore can result in acidification of affected tissues. Unsurprisingly, a role for pHRs, specifically GPR4, in regulating vessel growth has been described [26, 67, 92]. Intriguing new regulatory insight has now been gained regarding GPR4, which was shown to be associated with GPCR associated sorting protein 1 (GPRASP1) [45]. Loss-of-function mutations in GPRASP1 led to activation of GPR4 signalling pathways, which in turn promoted aberrant vessel formation [45]. Furthermore, loss of GPR4 function has been linked to lack of acidosis-mediated growth of new blood vessels in patients with coronary artery disease, who display lower GPR4 expression levels in endothelial progenitor cells than healthy individuals [60].

Whilst an essential component of all blood vessels is endothelia, other cell types are also involved in regulating vasculature function. Smooth muscle cells surround endothelial cells (except in capillaries and pericytic venules) and their function is to increase or decrease the vessel diameter which in turn adjusts blood pressure to the desired level. TDAG8 has been implicated in atherosclerosis, a condition in which the blood vessel diameter is obstructed by plaque build-up. TDAG8 is proposed to accelerate progression of this condition in a high-fat diet fed ApoE mouse model, by promoting vascular smooth muscle cell proliferation and migration. The latter effects were also observed in cultures of human vascular smooth muscle cells [9], suggesting that results obtained for TDAG8 in the murine atherosclerosis model may be translatable into humans.

## Lung

Proper lung function requires airways to be covered by surface liquid, the pH of which is tightly regulated in healthy individuals and shows aberrant values in several diseases including cystic fibrosis and bronchitis [99]. Considerable progress has been made in our understanding of PARs in lung function. Expression of TDAG8 and OGR1 was increased in a murine model of allergic asthma in bronchial smooth muscle cells. Intriguingly, this study also found that human bronchial smooth muscle cells only express TDAG8 and OGR1 whilst murine bronchial smooth muscle cells also express GPR4 [10]. Furthermore, TDAG8 was shown to be upregulated in lung fibroblasts from patients with idiopathic pulmonary fibrosis compared to healthy individuals, whilst OGR1 was downregulated in these cells, and GPR4 was not detected in primary human lung fibroblasts [65], hence mirroring expression patterns of bronchial smooth muscle cells.

OGR1 has been implicated in allergen-induced hyper-responsiveness and idiopathic pulmonary fibrosis [3, 10, 56, 57] and was reported to be pro-inflammatory [29, 56]. It is expressed in lung fibroblasts [3, 56] and bronchial smooth muscle cells [10, 29]. Whilst OGR1 expression is increased in bronchial smooth muscle cells in an ovalbumin antigen-challenged murine asthma model [10], it is downregulated in fibroblasts from patients with idiopathic pulmonary fibrosis [65], which promotes myofibroblast differentiation, suggesting that OGR1 has anti-fibrotic effects in lung fibroblasts [56]. Since inflammation and fibrosis go hand in hand, this makes it difficult to assess the impact of targeting OGR1 in inflammatory lung disease, especially since the pro-inflammatory and anti-fibrotic pathways have both been suggested to be mediated by Gs-dependent signalling pathways following OGR1 stimulation [3, 57].

Importantly, OGR1 is overexpressed in bronchial smooth muscle cells from asthma patients, and mechanical stress typical of that occurring in lung tissue during asthma can induce disease-specific gene expression patterns [32]. Given that OGR1 is a coincidence detector for both extracellular protons and mechanical stress [90] and that OGR1 can induce gene transcription (e.g. [22]), there may be a pivotal role for OGR1 in translating mechanical stresses into gene expression, thus exacerbating the disease and its progression. However, as always, the fact that expression levels of a particular protein are changed in certain pathologies is not evidence for critical involvement of this protein in the development and/or progression of that pathology. Only functional studies can reveal causal links between proteins and physiological and/or pathological processes.

## Skin

Skin pH is well-known to be acidic, and skin pathologies also impact skin pH [27]. Alkaline skin pH is observed in chronic wounds that are slow to heal, suggesting a link between pH and ability for skin to repair itself [28]. A role for pHRs in skin is somewhat under-researched but there has been some progress over the past few years, in particular for TDAG8. CO_2_ was shown to inhibit UVB-induced inflammatory responses in human neonatal foreskin cells (HEKn cell line) by activating TDAG8, suggesting an anti-inflammatory role for TDAG8 [74]. Furthermore, of all pHRs, TDAG8 is most highly expressed in these cells, with OGR1 having 0.5 expression of TDAG8 expression at mRNA level and GPR4 levels being negligible [74]. TDAG8 ko then confirmed its involvement in combating skin inflammation though it was not shown whether or not percutaneous administration of CO_2_ resulted in pH changes [74]. A follow-on report then found that mild pH decreases due to transcutaneous administration of CO_2_ gave rise to increased extracellular matrix production as well as increased TGF-β1 production in normal human dermal fibroblasts that was at least in part due to activation of both GPR4 and TDAG8, suggesting a role for these receptors in wound repair [84].

Additionally, genome-wide association studies show that TDAG8 intronic SNP rs8005161 variant, which shows reduced TDAG8 signalling ability, has a strong association with atopic dermatitis (in addition to inflammatory bowel diseases and asthma) [93]. Genome-wide *TDAG8* ko in an MC903 model of atopic dermatitis resulted in stronger disease progression than in wt, and CD4^+^ T cells from rs8005161 heterozygous humans had higher TNFα levels than individuals with reference *TDAG8* sequence [93]. All these findings support a role for TDAG8 in reducing skin inflammation and—together with GPR4—in promoting skin repair.

## Kidneys

One of the many functions of kidneys is blood pH homeostasis, and deviations in blood pH from the physiological range can affect kidney functions. Metabolic acidosis is accompanied by increased urinary Ca^2+^ excretion [1]. OGR1 has now been shown to be responsible for this by upregulating the function of NHE3, a sodium proton exchanger [24]. This is the first report providing a mechanistic explanation for acidosis-mediated renal Ca^2+^ excretion [24]. Subsequent experiments demonstrated that, in an oxalate-induced murine model of crystalline nephropathy, *OGR1* (but not *GPR4*) deficiency led to impaired kidney function due to increased Ca^2+^ oxalate deposition in kidney tubules [97]. Hence, OGR1 is emerging as an important player in renal disease and a promising target for treating unwanted effects of metabolic acidosis.

## Gastrointestinal (GI) tract

An intriguing feature of the GI tract is that its pH milieu changes from oral to aboral and from its lumen through the mucosal layer [4], with the largest variability observed in the small and large intestine, where pH values can range between pH 5 and pH 8 [34]. The intestinal pH is influenced by its microbiota [75], which in term is affected by lifestyle choices, disease burden and genetic factors of an individual [62]. A role for pHRs in inflammatory bowel diseases is well established [23, 25, 100], and the past few years have seen several papers investigating a role for TDAG8 in GI function. One study compared three mouse strains for differences in genes that may confer susceptibility to chemically induced colitis and identified *TDAG8* as one of five lead candidates [40]. Consistent with *TDAG8* polymorphisms influencing propensity for GI disease, the *TDAG8* I231L polymorphism, which reduces TDAG8 cAMP signalling ability [7], renders mice more susceptible to bacteria-induced colitis by increasing pro-inflammatory behaviour of immune cells and hence promoting intestinal inflammation [7, 52]. Intriguingly, TDAG8 expression is reduced in macrophages derived from monocytes isolated from patients with acute monocytic leukaemia, and this appears to negatively influence bacterial phagocytosis by these cells [52].

Furthermore, TDAG8 appears to play a role in regulating the gut microbiome since intestinal epithelial cell-specific deletion of *TDAG8* abolished homeostatic antimicrobial programs from these cells, thus rendering mice more prone to colitis [58]. Finally, *TDAG8* ko not only increased intestinal inflammation and fibrosis in a chemically induced mouse model but also the chances of developing colorectal cancer [51]. All these findings suggest that functional TDAG8 promotes gut health. Yet, one study found that TDAG8 has the potential to enhance gut inflammation by promoting differentiation of CD4^+^ cells into T helper 1 and T helper 17 cells [46]. Hence, TDAG8 has been allocated pro- and anti-inflammatory properties in the GI tract. However, it is important to consider that genome-wide knock out of *TDAG8* or *TDAG8* polymorphisms that reduce its signalling ability impact all cells in the affected individual. Hence, the pro-inflammatory effect of TDAG8 should be as compromised as its anti-inflammatory effects. The fact that under conditions of genome-wideTDAG8 impairment individuals are still more prone to developing inflammatory intestinal diseases may suggest that compromised T helper cell differentiation does not play a key role in GI tract inflammation development and progression.

## Musculoskeletal system

The impact of pHRs in inflammatory diseases other than bowel diseases has also been addressed in the past few years, and in particular the musculoskeletal system has received increased attention. Both osteoarthritis (which arises from mechanical wear and tear of joints) and rheumatoid arthritis (an autoimmune disease of joints) have been investigated with a view to pHR involvement.

GPR4 is an emergent player in osteoarthritis. It is overexpressed in cartilage of patients affected by this condition [43] and upregulated the expression of matrix-degrading enzymes and inflammatory factors [43, 47]. Moreover, overexpression of GPR4 accelerated the development of posttraumatic and ageing-associated osteoarthritis in mice whilst GPR4 ko had the opposite effect [43]. Consistent with a role for GPR4 in the development of osteoarthritis is the finding that advanced glycation end products, which have been linked to osteoarthritis [73], increase GPR4 expression [47].

Furthermore, consistent with a role for GPR4 in use-dependent degeneration in the musculoskeletal system, it has been implicated in intervertebral disc degeneration, where it is proposed to both progress the disease and contribute to the ensuing pain [44]. Finally, GPR4 has also been linked to rheumatoid arthritis by affecting synovial mast cell function [42].

In addition to GPR4, TDAG8 has also been implicated in rheumatoid arthritis. In a chemically induced mouse model of rheumatoid arthritis, *TDAG8* deletion was shown to prevent an increase in satellite glial cells and pro-inflammatory macrophages observed in the rheumatoid arthritis mouse model and appeared to reduce mechanical and thermal hyperalgesia and arthritis scores in the affected animals [16]. A subsequent study went on to demonstrate that the chemically induced rheumatoid arthritis mouse model exhibited alterations in their gut microbiome that could be reversed by TDAG8 inhibition [58]. This finding is very intriguing because pathological alterations of the gut microbiome have been implicated in immune system dysfunction and could therefore be a contributing factor to rheumatoid arthritis [13]. It is also the second study to imply TDAG8 in regulating the gut microbiome. Finally, genome-wide association studies have linked *TDAG8* with ankylosing spondylitis, a type of arthritis that causes inflammation of the joints and ligaments of predominantly the spine [2, 15].

Taken together, all results so far point towards a disease-promoting role of GPR4 and TDAG8 in the musculoskeletal system.

Whether or not OGR1 plays a role in bone metabolism remains one of the enduring mysteries. Different studies using genome-wide OGR1 knockout yield distinct results. However, one inherent issue with genome-wide knockouts is the potential for compensation, which is generally not addressed. Another issue is that effects of loss of gene expression may only become apparent when a particular tissue is stressed, either by unfavourable physiological conditions or by inducing pathologies. In this context, two studies that address potential roles for OGR1 in bone tissue using cell-type specific knockout approaches are of interest. Using an osteoclast-specific OGR1 knockout mouse, the impact of OGR1 deletion from these cells on bone metabolism was investigated under physiological conditions and in the presence of metabolic acidosis. This study demonstrated that OGR1 is essential for osteoclast function and in particular for bone resorption [36]. These findings were then extended in a second study that sought to address potential differences in roles for OGR1 in osteoclasts and osteoblasts. Using osteoblast- and osteoclast-specific OGR1 knockout, this study demonstrated that OGR1 stimulation in osteoblasts and osteoclasts resulted in cell-type specific responses under conditions of metabolic acidosis (osteoblasts: *cox2*, *fgf23* gene expression; osteoclasts: mineralisation and alkaline phosphatase activity as well as osteoclast-specific gene expression) that both contribute different aspects to OGR1-mediated bone loss under conditions of metabolic acidosis [37].

## Cancer

Cancer was one of the first diseases in which pHRs were implicated. Many different cancers have been investigated in different experimental models, from isolated cells to whole animal experiments, and it remains difficult to find patterns in the role that pHRs play in cancer development and progression. Research published in the past few years adds to the complicated picture. Several studies have investigated OGR1 in cancer, specifically in pancreatic, breast, ovarian, skin, liver, head and neck, colorectal and oesophageal cancer [5, 6, 14, 18, 30, 33, 48, 54, 63, 77, 78, 88, 96, 98, 101], ranging from pure expression (e.g. [5, 48]) to functional studies in cells (e.g. [77, 96, 98]) and/or mice (e.g. [6, 54, 98]). As before, results do not seem to show any clear patterns: OGR1 was found to be involved in processes that promote or interfere with cancer progression [6, 14, 17, 30, 54, 63, 77, 78]. Discrepancies may arise because some studies address a role for OGR1 in the host (tissue) whilst others look at OGR1 roles in cells making up the tumour tissue. A further complication could arise from the fact that OGR1 may act in a sex-dependent manner. Melanoma growth in response to injecting the murine melanoma cell line B16F10 into wildtype and *OGR1* ko mice of both sexes resulted in reduced tumour growth only in male but not female *OGR1* ko mice [98]. This was then shown to be a consequence of reduced immune cell infiltration in the ko male animals [98]. Importantly, results using the same cell line and male only mice with a different background had previously shown that *OGR1* ko mice exhibit reduced tumour growth due to impaired immune cell function in the host animals [6]. In contrast, a study using only female mice and the squamous carcinoma cell line 7 (a murine oral cancer cell line) found that OGR1 supported cancer progression by inhibiting immune cell function [54]. Specifically, it was shown that OGR1 (and TDAG8) increased expression of programmed cell death protein 1, which anergises T cells, thus preventing an immune response and enabling cancer growth and progression [54].

There is one important development that has the potential to affect many distinct cancers. Whole genome sequencing identified 51 rare variant carriers that were significantly associated with peripheral neuropathy, a common side effect of chemotherapy [31]. Of these, two C-terminal *OGR1* variants were shown to be a risk factor for chemotherapy-induced peripheral neuropathy, possibly due to alterations in arrestin binding and subsequent aberrant activation of OGR1 in so-called PEP1 neurons, a subgroup of C-fibre nociceptors that express a specific subset of proteins and play a key role in pain sensation [31]. This is a significant finding because it may enable the identification of patients who are more likely to develop neuropathies as well as open up the possibility of treating neuropathies in patients by developing pharmaceutical inhibitors of this *OGR1* variant.

## Reproduction

Exciting discoveries have been made in relation of pHR involvement in reproduction, an area of research that has received very little attention to date. pHRs have now been implicated at different levels of the regulation of reproduction, from regulation of sex hormone release to placenta function.

GPR4 appears to be involved in hormone release from the anterior pituitary. Proton-mediated GPR4 activation in a pituitary cell line was shown to increased growth hormone and prolactin secretion [55], whilst a transcriptome analysis of gonadotropin releasing hormone (GnRH) neurons isolated from mice at different stages of their oestrous cycle showed a significant downregulation of GPR4 during the first half of the reproductive cycle [86]. These findings are intriguing since GnRH and prolactin have opposite effects on the release of luteinizing hormone from the anterior pituitary, which is essential for triggering ovulation in cyclic ovulators such as humans. These studies suggest that GPR4 activity could inhibit luteinizing hormone release from the pituitary, thus preventing ovulation.

Two other studies have investigated a role for GPR4 in trophoblasts and find that GPR4 expression was upregulated in preeclampsia (PE) placentas compared to healthy placentas [64, 66]. This may be a consequence of hypoxic and acidic conditions [66] that are characteristic for PE placentas [68]. GPR4 inhibited proliferation and migration of a trophoblast cell line [64] though given its role in vessel growth, a more pertinent role for GPR4 may lie in the abnormal vessel formation observed in PE placentas [68]. Given that preeclampsia occurs in 8% of all pregnancies with potentially devastating consequences, it seems that genetic variants of GPR4 are worthwhile studying in the context of preeclampsia.

GPR4 is not the only pHR that has been implicated in placental function. TDAG8 was shown to inhibit trophoblast cell adhesion, invasion and growth under acidic conditions, suggesting that activation of TDAG8 reduces chances of successful implantation of the conceptus [50]. Consistent with this, TDAG8 was more highly expressed in villous tissue during early pregnancy loss [50]. Finally, *TDAG8* is one of 18 candidate genes in chicken that regulate egg production [8]. All these findings suggest that pHRs may have important roles to play in various aspects of reproductive function.

## Open questions and outstanding issues

There are a number of important and pertinent open questions and outstanding issues regarding pHRs, many of which have already been raised and will therefore not be repeated here [25, 81].

One additional exciting question is whether pHRs influence their own and/or each other’s activity. pHRs can change extracellular pH by affecting proton transport across membranes (e.g. [11, 53]). Hence, they have the capacity to self-regulate and regulate each other. The extent to which this happens and whether this affects physiological function and/or disease progression remains unexplored but may have important therapeutic implications.

OGR1 is particularly interesting in this context because it requires both an increase in extracellular proton concentration and a certain level of cellular actin polymerisation to be active [90]. pHR activation can not only alter extracellular pH but also affect the polymerisation state of the actin cytoskeleton of cells directly, by inducing stress fibre formation (e.g. [50, 85]), and indirectly, by changing the extracellular matrix composition (e.g. [43, 47, 84]). The extracellular matrix in turn affects the actin polymerisation state of surrounding cells since cells change their shape to accommodate altered mechanical properties of their surroundings [12]. Crucially, changes in extracellular matrix composition, like changes in extracellular pH, accompany virtually all pathologies [23, 41]. Hence, signalling through OGR1 can change either when extracellular pH changes or when the extracellular matrix changes. Hence, OGR1 is predisposed to measure pathological tissue changes, since its activity increases with extracellular acidification and matrix stiffening. This would in turn suggest that, conceivably, low OGR1 signalling activity is a sign of healthy cells and tissues and that OGR1 may have a surveillance role.

To date, OGR1 is the only pHR for which a natural human functional ko has been shown to be causal for a pathology, amelogenesis imperfecta. The link between OGR1 and aberrant enamel formation was first established in 2016 [61] when three families with amelogenesis imperfecta type IIA6 (i.e. amelogenesis imperfecta caused by hypomineralisation) were shown to have homozygous genetic variants of *OGR1* that resulted in loss of function of OGR1 protein. Importantly, no other abnormalities were reported [61]. More recently, a new family has been identified with a homozygous frameshift mutation in *OGR1*, resulting in a truncated OGR1 protein, and again only amelogenesis imperfecta is reported [76], and a whole genome sequencing study investigating natural human kos identified an individual who was homozygous for the already reported nonsense variant NM_003485.3:c.1006G > T of *OGR1* [82].

Intriguingly, however, a different genome-wide association study looking into causes for osteoporosis identified *OGR1* as a gene that is “associated with … Mendelian diseases with high impact on bone strength, or associated with bone mineral density or fracture risk …” [69]. An individual with a heterozygous *OGR1* exonic deletion was identified, who was described as healthy but having suffered a “vertebral fracture during light physical activity” [69]. This study is a first correlation between OGR1 loss-of-function and osteoporosis in humans, and it ties in very nicely with the studies investigating impact of osteoclast- and osteoblast-specific OGR1 knockout discussed above [36, 37]. However, neither *OGR1* ko mice nor human individuals with functional deletion of *OGR1* have been reported to have defects in bone mineralisation. The main difference here is the allelic extent of the *OGR1* deletion, which is heterozygous in the case of the individual with osteoporosis [69] and homozygous in the other cases. Hence, it may be that OGR1 expression relative to other proteins is important in bone metabolism, and that homozygous, but not heterozygous, loss of function can be compensated for in bone. Whether enamel formation was also affected in the heterozygous individual is unclear since the state of teeth was unreported [69]. It is therefore worthwhile investigating whether heterozygous and homozygous *OGR1* loss affect physiological processes differently.

Since loss of function of OGR1 does not appear to interfere with normal body functions (apart from enamel mineralisation, which is completed around age seven in humans), this makes OGR1 an excellent pharmaceutical target. Off-target effects would be unlikely or only minor so that only few side effects would be expected when inhibiting or stimulating OGR1 to suppress unwanted processes in affected cells and tissues.

Finally, it is worth noting that the terminology around proton-sensing needs clarification. As stated above, the function of any protein is affected by pH because its conformation is in part dependent on hydrogen bonding. Hence, it would be helpful to introduce a set of conditions under which a protein is considered activated (rather than allosterically modulated) by protons. These conditions could include the presence of His residues that are demonstrated to respond to (extracellular) protons and whose elimination therefore impacts activation of the receptor. Another criterion could be a low proton concentration requirement for activation, to ensure specificity of the response. However, problems will arise from these definitions since His residues are not the only amino acids capable of proton-sensing (see [70]), and because the function of some proton sensors may be to report only extreme pH values rather than relatively small fluctuations.

pHRs are starting to receive the attention that they deserve. They are a fascinating group of receptors whose potential is only just being discovered, and the coming years will see a great expansion of our knowledge of these receptors and their biological roles.

## Data Availability

No datasets were generated or analysed during the current study.
